# Distinguishing epigenetic marks of developmental and imprinting regulation

**DOI:** 10.1186/1756-8935-3-2

**Published:** 2010-01-15

**Authors:** Kirsten R McEwen, Anne C Ferguson-Smith

**Affiliations:** 1 Department of Physiology, Development and Neuroscience, University of Cambridge, Downing Street, Cambridge CB2 3EG, UK

## Abstract

**Background:**

The field of epigenetics is developing rapidly, however we are only beginning to comprehend the complexity of its influence on gene regulation. Using genomic imprinting as a model we examine epigenetic profiles associated with different forms of gene regulation. Imprinting refers to the expression of a gene from only one of the chromosome homologues in a parental-origin-specific manner. This is dependent on heritable germline epigenetic control at a *cis*-acting imprinting control region that influences local epigenetic states. Epigenetic modifications associated with imprinting regulation can be compared to those associated with the more canonical developmental regulation, important for processes such as differentiation and tissue specificity. Here we test the hypothesis that these two mechanisms are associated with different histone modification enrichment patterns.

**Results:**

Using high-throughput data extraction with subsequent analysis, we have found that particular histone modifications are more likely to be associated with either imprinting repression or developmental repression of imprinted genes. H3K9me3 and H4K20me3 are together enriched at imprinted genes with differentially methylated promoters and do not show a correlation with developmental regulation. H3K27me3 and H3K4me3, however, are more often associated with developmental regulation. We find that imprinted genes are subject to developmental regulation through bivalency with H3K4me3 and H3K27me3 enrichment on the same allele. Furthermore, a specific tri-mark signature comprising H3K4me3, H3K9me3 and H4K20me3 has been identified at all imprinting control regions.

**Conclusion:**

A large amount of data is produced from whole-genome expression and epigenetic profiling studies of cellular material. We have shown that such publicly available data can be mined and analysed in order to generate novel findings for categories of genes or regulatory elements. Comparing two types of gene regulation, imprinting and developmental, our results suggest that different histone modifications associate with these distinct processes. This form of analysis is therefore a useful tool to elucidate the complex epigenetic code associated with genome function and to determine the underlying features conferring epigenetic states.

## Background

Epigenetic mechanisms play an important role in the control of gene expression. Modification to the packaging of DNA is believed to allow a more open or closed structure and influences association of the transcriptional machinery with the genetic material. The most characterised examples of epigenetic mechanisms to date in mammalian cells include DNA methylation of cytosine and post-translational modifications to the core histone proteins of the nucleosome (reviewed in [1]), though other epigenetic mechanisms are known to exist. Nevertheless, little is known regarding exactly how these two processes act to regulate gene expression.

The transcriptome of a cell is tightly regulated by epigenetic mechanisms to allow correct gene expression patterns at appropriate time points. The dynamic changes in gene expression required during the proliferation, differentiation and commitment of specific cell types are associated with specific epigenetic alterations. In order to simplify the description of this type of regulation we shall hereafter refer to this as developmental regulation.

An additional mechanism of gene regulation is that of genomic imprinting, an epigenetic process affecting less than 1% of genes in the mammalian genome. An imprinted gene refers to a gene in which expression occurs solely or predominantly from only one of the parental chromosome homologues (reviewed in [2]). Either the gene copy inherited from the mother is active while the paternal copy is inactive, or vice versa in the case of a different imprinted gene. In order to achieve this functional haploidy at selected genes, epigenetic mechanisms are utilised to differentiate between the genetically identical sequences and confer monoallelic activity.

Both DNA methylation and post-translational histone modifications have been found to be enriched to a greater degree on one chromosome compared to its homologue at a number of imprinted loci in the mouse and human (reviewed in [3,4]). Differential DNA methylation between the two parental chromosomes is found at many (though not all) imprinted gene promoters, where methylation is present on the inactive allele (reviewed in [3]). This can either be established in the germline or post-fertilisation (defined, respectively, as germline and somatic differentially methylated regions; DMRs). Germline DMRs are found at all imprinting control regions (ICRs); deletion of an ICR leads to the disruption of imprinting of nearby imprinted genes, demonstrating the fundamental nature of these elements in imprinting regulation (reviewed in [3]).

Many studies of specific loci have described differential enrichment of particular histone modifications between the two parental chromosomes at some ICRs and imprinted gene promoters or transcription start sites (TSSs). These marks include histone H3 acetylation, H4 acetylation, H3 dimethylation at lysine 4 (H3K4me2) and H3K4me3, which are found preferentially enriched on the unmethylated chromosome or normally active allele in comparison with its homologous counterpart, and H3K27me2, H3K27me3, H3K9me2 and H3K9me3 preferentially enriched on the methylated chromosome or inactive allele [5-42]. H4K20me3 has previously been shown to be preferentially enriched on the methylated chromosome of eight ICRs [6,20,28,35,36,38,40]. At non-ICR regions, limited experimental analysis has been undertaken to test for enrichment of this mark - no preferential enrichment was found for one imprinted cluster [35] and data conflicted when different experimental approaches were used for one other gene [20]. Two non-allele-specific studies have identified a higher proportion of coenrichment of active (H3K4me2/3) and repressive (DNA methylation and H3K9me3) marks at imprinted loci compared to other loci, verifying the ability to detect histone modifications preferentially enriched on one of the two chromosomes using microarray platforms [43,44]. Importantly, individual genes and individual ICRs show different combinations of enriched histone modifications, with cell-type specificity also apparent. Despite many studies having previously assessed epigenetic modifications at particular imprinted genes, the functional role that histone modifications play in imprinting establishment and maintenance, or in relation to other processes at these unique genes, is difficult to assess and is currently unknown.

Key to the work presented here, the active alleles of imprinted genes are also developmentally regulated. Imprinted genes that are developmentally expressed have only the 'normally active' allele expressed, whereas developmentally repressed imprinted genes have the 'normally active' allele repressed resulting in two repressed alleles. Very few studies have assessed the nature of epigenetic marks at developmentally repressed imprinted genes. We have used imprinted genes as a model system to compare epigenetic marks associated with the control of imprinting to those associated with developmental regulation to test the hypothesis that distinct epigenetic modifications are employed for these two mechanisms.

An increasing number of groups are describing the epigenetic characteristics of various cell types across the whole genome in both mouse and human and these studies produce a large amount of publicly accessible data (see Additional file 1: Chromatin and expression states; also reviewed in [1]). These studies generate huge datasets allowing general functional correlates to be made. For example, H3K4me3 is associated with expressed genes and H3K27me3 with repressed genes [45]. Also, H3K9me3 is present at inactive regions of the genome, such as constitutive heterochromatin, where it is found along with H4K20me3 [45]. A few reports have identified H3K9me3 within actively transcribed regions of the genome, however this modification has not been generally found enriched at the TSS of expressed genes [45-48]. Further insight into epigenetic mechanisms can be gained through the exploration of specialised processes such as genomic imprinting.

The data from high-throughput studies has been used here as a source to extract and subsequently analyse epigenetic profiles found at all imprinted genes in embryonic stem cells (ESCs) and in more differentiated cell types (see Additional file 1: Chromatin and expression states). We have determined from high-throughput expression data [49-51] that some imprinted genes are developmentally expressed while others are developmentally repressed in the characterised cell types (Additional file 1: Chromatin and expression states). Integration of the expression status and of the differential DNA methylation status with histone modification profiles has allowed us to distinguish histone modifications associated with imprinting from those more often associated with developmental repression. Our results have been considered alongside data from allele-specific histone modification studies of imprinted loci.

## Results

### Comprehensive analysis of epigenetic marks at imprinted genes

High-throughput studies assessing histone modification enrichment across mouse and human genomes in pluripotent and more differentiated cell types have been mined to characterise profiles of all known imprinted genes. Profiles of mouse and human imprinted genes confirmed at the time of analysis [52-54] (*n *= 97; see Additional file 1: Imprinted genes) were individually identified using data generated by six high-throughput studies [49-51,55-57] as a source (see Methods). Expression data was also assessed when included in the original study [49-51]. Each imprinted gene present in at least one study is listed in Additional file 1: Chromatin and expression states with its associated expression profile and/or histone modification profile in human ESCs, liver and pro-B cells (REH) in addition to mouse ESCs, neural progenitor cells (NPCs) and embryonic fibroblasts (MEFs). The histone modifications H3K4me3, H3K27me3, H3K9me3, H4K20me3, H3K36me3, H3K9/14ac and H3K79me2 were assessed predominantly across TSSs. The histone modification profiles of 64 mouse imprinted genes and 46 human imprinted genes confirmed to be imprinted in at least one tissue have been characterised.

### Imprinted genes show unique histone modification profiles in mouse ESCs

Through further analysis of data mined from high-throughput experiments, histone modification profiles common to imprinted genes can be determined. We have assessed all imprinted genes for enrichment of three histone modifications, H3K4me3, H3K27me3 and H3K9me3, in mouse ESCs utilising source data from Mikkelsen *et al*. [50]. Comparing the enrichment patterns of these marks at imprinted genes to all other genes within the mouse genome highlights whether imprinted genes are epigenetically regulated in a unique manner.

**Figure 1:**
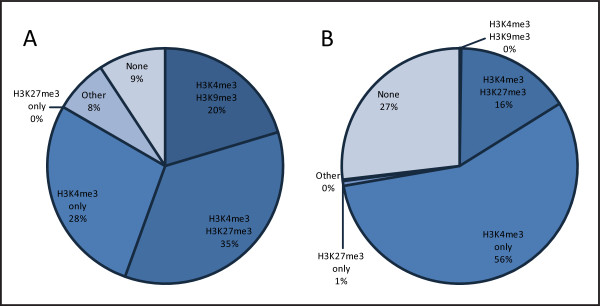
**Histone modification patterns in mouse embryonic stem cells (ESCs)**. Profiles for H3K4me3, H3K27me3 and H3K9me3 were analysed in mouse ESCs at transcription start sites of (A) imprinted genes (*n *= 54) and (B) all genes (*n *= 17,761) using source enrichment data of Mikkelsen *et al*. [50]. 'Other' represents alternative combinations of these three histone modifications and 'None' represents genes without any of these three modifications. The data does not meet the conditions of the chi-square test when comparing all profiles and also when specifically comparing H3K4me3 and H3K9me3 between imprinted and all genes. H3K4me3 and H3K27me3 are together enriched more often at imprinted genes than all genes (Yates' chi-square test, *P *< 0.005).

Figure 1 illustrates the enrichment profiles for imprinted genes and for all genes in the mouse genome and identifies two striking differences. Firstly, a higher number of imprinted genes are marked by both H3K4me3 and H3K9me3 relative to all mouse genes (20%, *n *= 54 compared to 0%, *n *= 17,761 respectively). Secondly, 35% of imprinted genes are enriched with both H3K4me3 and H3K27me3 compared to 16% of all genes in the mouse genome (*n *= 54 and *n *= 17,761 respectively; Yates' chi-square test, *P *< 0.005). We discuss the H3K4me3/H3K9me3 profile below; whether the H3K4me3/H3K27me3 profile reflects developmental or imprinting regulation is addressed subsequently.

As H3K9me3 enrichment has previously been linked to H4K20me3 [45], we also assessed enrichment of H4K20me3 at imprinted genes in mouse ESCs. Regions throughout the genome have been defined as enriched with H4K20me3 by Mikkelsen *et al*. [50] using a different, more stringent method to that used to define enrichment of the other three histone marks (see Methods), therefore this mark may be slightly underrepresented in comparison. Interestingly, at all but one of the imprinted gene TSSs enriched with both H3K4me3 and H3K9me3 in ESCs, H4K20me3 is also present (data not shown). Furthermore, all of the imprinted genes enriched with these three histone modifications have a DMR established in the germline present at the gene promoter. A number of germline DMRs in the mouse have previously been experimentally deleted and, as a result, all but one of these have been confirmed to act as the ICR at the respective imprinted locus (reviewed in [3]). We therefore subsequently assessed all known ICRs and identified enrichment peaks for all three histone modifications not only at all ICRs located at gene promoters, but also at all ICRs located at intergenic regions (data not shown). H3K27me3 was not found at all of the known ICRs. Intriguingly, the one germline DMR known *not *to act as an ICR (*Gnas Exon1A*) does *not *exhibit this specific tri-mark profile. These findings imply that the presence of H3K4me3, H3K9me3 and H4K20me3 is a true epigenetic signature of ICRs.

### H3K9me3 and H4K20me3 associate with imprinting rather than developmental regulation

**Figure 2:**
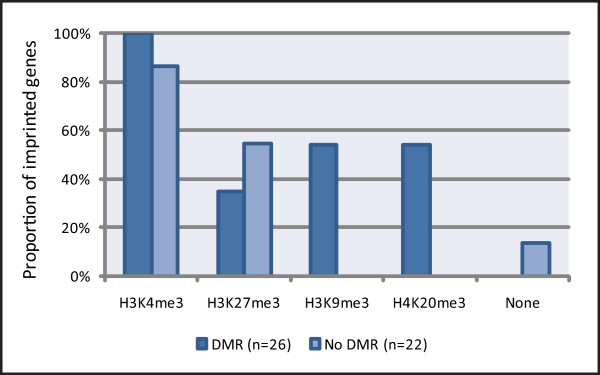
**Impact of promoter differential methylation on histone modification profiles at imprinted genes**. Histone modification enrichment is compared in mouse embryonic stem cells at imprinted genes with and without a promoter differentially methylated region (DMR). Transcription start sites were assessed for enrichment of H3K4me3, H3K27me3, H3K9me3 and H4K20me3 using source data from Mikkelsen *et al*. [50]. The presence of one particular modification at an imprinted gene does not preclude the presence of another. A significantly different epigenetic profile for these four marks is observed at genes with a promoter DMR compared to genes without a promoter DMR (chi-square contingency test, *P *< 0.0001). Germline DMRs are not distinguished from somatic DMRs in this analysis. H3K9me3 and H4K20me3 are exclusively enriched at imprinted genes possessing a promoter DMR. A greater proportion of genes with promoter DMRs are developmentally expressed compared to genes without promoter DMRs (71% compared to 48% respectively); the increase in H3K4me3 and the decrease in H3K27me3 at genes with a promoter DMR likely reflects this (see Additional file 2 and Figure 4).

In order to assess the histone modification enrichment profiles at both germline and somatic DMRs we have compared imprinted genes with and without promoter DMRs for enrichment of H3K9me3, H4K20me3, H3K4me3 and H3K27me3 in mouse ESCs using the same source data as above (Figure 2; for gene classifications see Additional file 1: Promoter DMR status). Imprinted genes with a promoter DMR (*n *= 26) have a significantly different epigenetic profile for these four marks to imprinted genes without a promoter DMR (*n *= 22) in mouse ESCs (chi-square contingency test, *P *< 0.0001).

As seen from Figure 2, H3K4me3 and H3K27me3 do not show obvious associations with promoter DMR status. However, H3K9me3 and H4K20me3 are exclusively present at imprinted genes that possess a promoter DMR. This most likely represents enrichment on the DNA methylated, inactive allele based on results of previous allele-specific studies as discussed above. Interestingly, when assessing the nature of the DMRs that have both H3K9me3 and H4K20me3 enriched (*n *= 12), we find that all are germline DMRs rather than somatic DMRs indicating that the combined presence of H3K9me3 and H4K20me3 only occurs in the presence of H3K4me3 specifically at germline DMRs, defining the identified tri-mark profile.

The 'normally active' allele of an imprinted gene can be developmentally regulated; we have therefore sorted mouse imprinted genes by expression status in ESCs using microarray data from Mikkelsen *et al*. [50]. This microarray study does not distinguish between the two parental alleles, however, the overall expression status can be considered to reflect the developmental expression status of the 'normally active' allele. This assumption will not hold true for a limited number of cases as some known imprinted genes do not display imprinting in ESCs (for example, the placentally-imprinted genes *Cd81*, *Osbpl5 *and *Tssc4 *are biallelically expressed in ESCs [10]). By integrating expression and histone modification profiles of imprinted genes we can assess the potential role of specific histone marks in developmental regulation (see Additional file 2; note that the presence of one particular modification at an imprinted gene does not preclude the presence of another).

If a particular modification is enriched to an equal degree at developmentally expressed and repressed imprinted genes, it is likely that this modification is *not *associated with developmental regulation, but rather with imprinting. Additional file 2 shows that H3K9me3 is approximately equally enriched at developmentally expressed and repressed imprinted genes. This supports our finding that this mark is more likely to be involved in allelic imprinting regulation than developmental regulation. H4K20me3 is not enriched to a greater degree at developmentally repressed imprinted genes compared to expressed genes which again is inconsistent with a role in developmental repression; in fact a higher incidence is observed at developmentally expressed compared to repressed genes (Additional file 2). This is a result of a higher number of genes that possess a promoter DMR being developmentally expressed (71% of genes with a promoter DMR are expressed compared to only 48% of genes without a DMR).

Histone modification enrichment at developmentally expressed or repressed imprinted genes can be assessed alongside the promoter DMR status. Of all developmentally repressed genes *without *promoter DMRs that were assessed, none are enriched for H3K9me3 or H4K20me3 (*n *= 11; data not shown). Together, our results strongly suggest that H3K9me3 and H4K20me3 are not associated with developmental repression of imprinted genes in mouse ESCs, but rather with imprinting control.

### H3K4me3 and H3K27me3 in developmental regulation of imprinted genes

The results depicted in Additional file 2 suggest a propensity for H3K27me3 to play a role in developmental repression of imprinted genes: in mouse ESCs H3K27me3 is enriched to a greater degree at developmentally repressed imprinted genes (53%) than at expressed genes (25%). In contrast, H3K4me3 is found at a high number of both developmentally expressed and repressed imprinted genes. An association of H3K4me3 enrichment with developmental and/or imprinting regulation is more difficult to distinguish due to the previous identification of H3K4me3 enrichment not only at expressed genes, but also at repressed genes when in combination with H3K27me3 (see below).

Our results in Figure 1 show that these two histone modifications are indeed coenriched at a large proportion of imprinted genes in mouse ESCs, showing a significant difference compared to all mouse genes. We have also assessed this profile using source data from two different published studies of human ESCs [51,56] and again observe a significant difference between imprinted genes and all other genes in the genome (24% of imprinted genes compared to 10% of all genes, Yates' chi-square test, *P *< 0.01 for [51] and 30% of imprinted genes compared to 19% of all genes, Yates' chi-square test, *P *< 0.05 for [56]; data not shown). Many different laboratories have shown that these two histone modifications coexist at some non-imprinted gene promoters, defined as a bivalent state [58]. This may correspond to genes with particularly dynamic developmental expression patterns. Imprinted genes often show complex expression profiles throughout development and may be more likely to exhibit bivalency, where both marks are present on the same developmentally regulated allele. Alternatively, or additionally, enrichment of both H3K4me3 and H3K27me3 at an imprinted gene may reflect enrichment of H3K4me3 on the active/developmentally regulated allele and H3K27me3 on the inactive allele.

**Figure 3:**
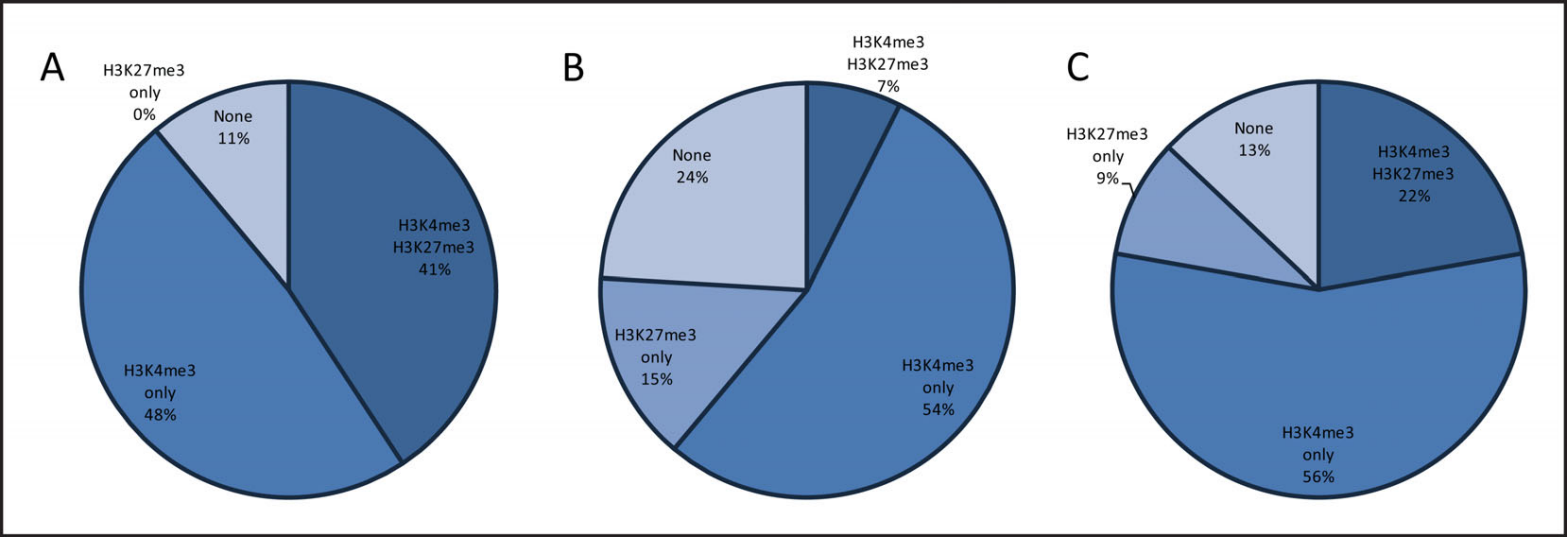
**Histone modification patterns at imprinted genes across cell types**. Histone modification profiles for H3K4me3 and H3K27me3 were analysed at transcription start sites of imprinted genes (*n *= 54) in mouse (A) embryonic stem cells (ESCs), (B) neural progenitor cells (NPCs) and (C) mouse embryonic fibroblasts (MEFs) using Mikkelsen *et al*. [50] whole-genome source data. The patterns of enrichment shown differ significantly across the three cell types (chi-square contingency test, *P *< 0.0005). Coenrichment of H3K4me3 and H3K27me3 is significantly higher at ESCs compared to NPCs (Yates' chi-square test, *P *< 0.01). A reduction from 41% in ESCs to 22% in MEFs is observed for this specific profile (Yates' chi-square test, *P *= 0.123).

In order to investigate this issue, we have characterised histone modification profiles for H3K4me3 and H3K27me3 in two more differentiated cell types, NPCs and MEFs, at the TSS of all imprinted genes using source data from Mikkelsen *et al*. [50] (Figure 3). The profiles shown differ significantly across the three cell types (chi-square contingency test, *P *< 0.0005). When comparing imprinted genes to all genes in the mouse genome enriched with both H3K4me3 and H3K27me3 a significant difference is observed for both NPCs and MEFs (7% of imprinted genes compared to 2% of all genes, Yates' chi-square test, *P *< 0.05 for NPCs and 22% of imprinted genes compared to 9% of all genes, Yates' chi-square test, *P *< 0.005 for MEFs; data not shown). A reduction in combined H3K4me3 and H3K27me3, but not complete removal, is observed between ESCs and the more differentiated cell types, suggestive of resolution of bivalency [41% in ESCs compared to 7% in NPCs (Yates' chi-square test, *P *< 0.01) and 22% in MEFs (Yates' chi-square test, *P *= 0.123) (Figure 3)]. An increase in the number of genes possessing only one of these two marks is also observed in NPCs and MEFs compared to ESCs. Also of note is the absence of H3K27me3 on its own (that is, without combined H3K4me3 enrichment) in ESCs.

**Figure 4:**
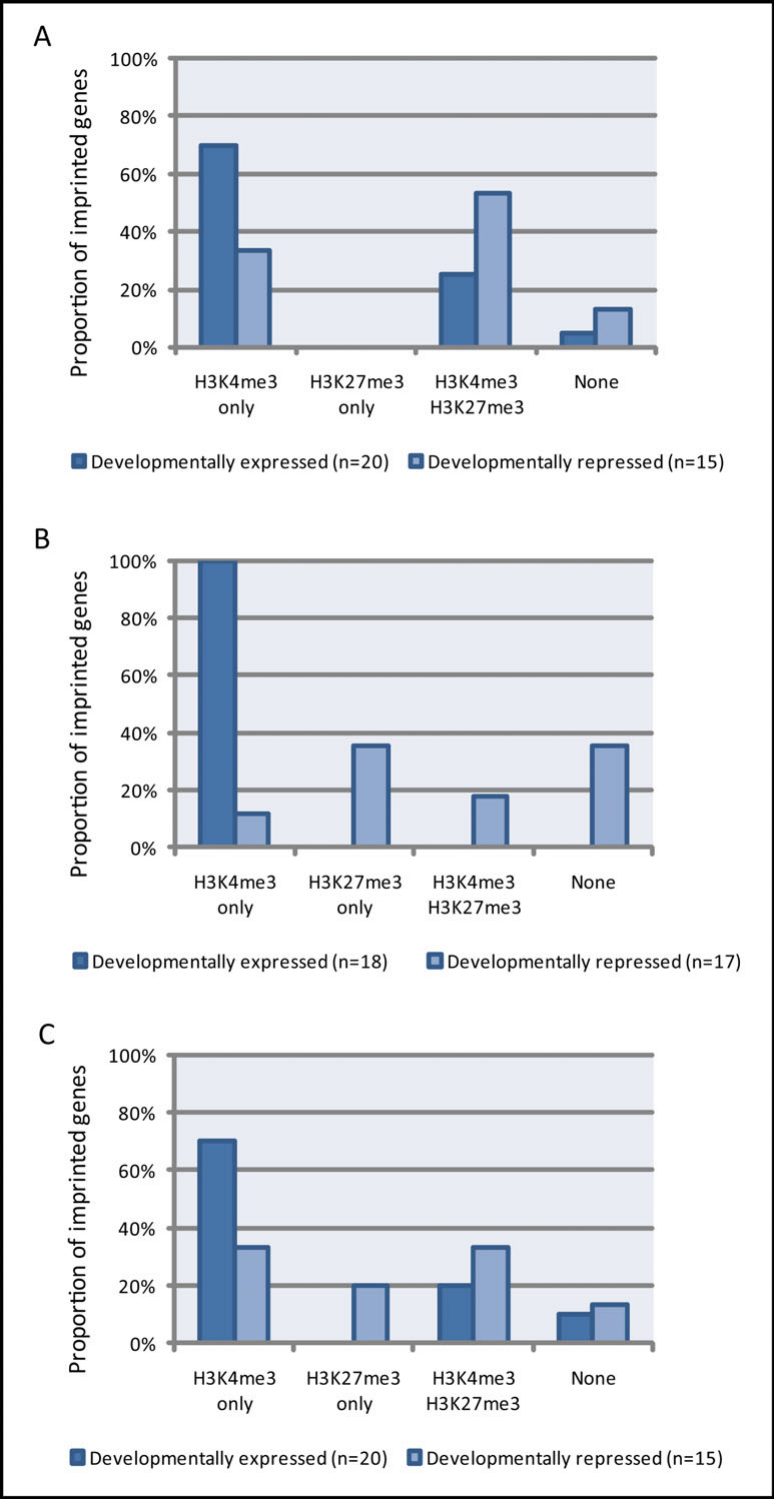
**Enrichment of H3K4me3 and H3K27me3 with respect to developmental expression status**. The two histone modifications H3K4me3 and H3K27me3 are shown to be important for the developmental regulation of imprinted genes. Using Mikkensen *et al*. [50] as a source for histone modification enrichment and expression data, histone modification profiles of H3K4me3 and H3K27me3 at imprinted gene transcription start sites were assessed in mouse (A) embryonic stem cells (ESCs), (B) neural progenitor cells (NPCs) and (C) mouse embryonic fibroblasts (MEFs) at developmentally expressed and repressed imprinted genes. Though a trend is observed, profiles at developmentally expressed imprinted genes do not differ significantly to repressed genes for ESCs or MEFs (chi-square contingency test, ESC:*P *= 0.097, MEF: *P *= 0.079). NPC data does not meet the conditions required for the chi-square contingency test.

In order to establish whether this does indeed reflect resolution of bivalent domains, we have assessed the presence of these marks both individually ('H3K4me3 only' and 'H3K27me3 only') and in combination (H3K4me3 and H3K27me3) with regard to developmental expression status (Figure 4). The combined enrichment of H3K4me3 and H3K27me3 is found at 53% of developmentally repressed imprinted genes whereas only 25% of expressed genes are coenriched in ESCs (Figure 4A). This implies that this particular histone modification profile is associated with developmental repression and, therefore, that these two modifications often mark the developmentally regulated, 'normally active' allele when repressed. Previous literature has found that non-imprinted genes showing bivalency are not expressed at high levels [59], which is consistent with our analysis.

Of the few genes that possess both marks in NPCs and MEFs, we observe a somewhat higher number of developmentally repressed imprinted genes with coenrichment than expressed genes (18% and 0% in NPCs, 33% and 20% in MEFs, respectively; Figure 4B and Figure 4C), consistent with our findings in ESCs. In NPCs and MEFs a decrease is observed compared to ESCs in the number of developmentally repressed imprinted genes with both H3K4me3 and H3K27me3, with an accompanying increase in the number of repressed genes with only H3K27me3. This further suggests resolution of the bivalent state and consequently supports involvement of these histone modifications in developmental regulation of imprinted genes. When looking specifically at the genes in ESCs that have H3K4me3 and H3K27me3 coenrichment and are developmentally repressed, enrichment is resolved to H3K4me3 in the more differentiated cell types upon gene activation in some, albeit not all cases (Table 1, upper panel). We also observe resolution of H3K4me3 and H3K27me3 coenrichment to H3K27me3 only from ESCs to NPCs and MEFs at some genes whose expression remains repressed (Table 1, lower panel). This bivalency may reflect the unique, adaptable structure of chromatin in pluripotent ESCs [60].

**Table 1:** Dynamics of H3K4me3 and H3K27me3 profiles at imprinted genes between cell types.

	ESCs		More differentiated cells		
		
*Gene*	*Expression*	*Histone modification*	*Expression*	*Histone modification*	*Cell type*
*Gatm*	Absent	H3K4me3 H3K27me3	Present	H3K4me3	NPC
			Present	H3K4me3 H3K27me3	MEF
*Peg12*	Absent	H3K4me3 H3K27me3	Present	H3K4me3	NPC+MEF
*Tfpi2*	Absent	H3K4me3 H3K27me3	Present	None	MEF

*Ascl2*	Absent	H3K4me3 H3K27me3	Absent	H3K4me3 H3K27me3	NPC+MEF
*Calcr*	Absent	H3K4me3 H3K27me3	Absent	H3K27me3	NPC+MEF
*Kcnq1*	Absent	H3K4me3 H3K27me3	Absent	H3K27me3	NPC+MEF
*Rasgrf*	Absent	H3K4me3 H3K27me3	Absent	H3K4me3 H3K27me3	NPC+MEF
*Slc22a3*	Absent	H3K4me3 H3K27me3	Absent	H3K27me3	NPC+MEF
*Tfpi2*	Absent	H3K4me3 H3K27me3	Absent	None	NPC

Expression and histone modification profiles at developmentally repressed imprinted gene transcription start sites (TSSs) enriched with both H3K4me3 and H3K27me3 in mouse embryonic stem cells (ESCs) are detailed for neural progenitor cells (NPCs) and mouse embryonic fibroblasts (MEFs). The presence of these two marks in ESCs sometimes resolves to H3K4me3 in the more differentiated cell types at genes that become expressed (upper panel). Resolution (to H3K27me3) also occurs at some genes that do not change expression status and remain repressed (lower panel). The expression status and histone enrichment profile at the TSS of imprinted genes were identified using source data from Mikkelsen *et al*. [50].

Enrichment of H3K4me3 without H3K27me3 ('H3K4me3 only') across all cell types is significantly greater at developmentally expressed than repressed imprinted genes (chi-square test, *P *< 0.01; Figure 4). 'H3K27me3 only' shows an association with developmental regulation in an opposing manner to H3K4me3, where it is only enriched at developmentally repressed imprinted genes for NPCs and MEFs (35% and 20% respectively). This is consistent with previous epigenetic studies assessing general associations of these marks with gene expression (reviewed in [1]).

### H3K4me3 and H3K27me3 occasionally reflect imprint status

We have shown above that H3K4me3 and H3K27me3 are associated with the developmental regulation of imprinted genes. However, enrichment of H3K4me3 without H3K27me3 is also observed at 33%, 12% and 33% of developmentally *repressed *genes in ESCs, NPCs and MEFs, respectively (Figure 4). This likely marks the 'normally active' allele, reflecting a memory of the imprint status of the gene. Hence, H3K4me3 is predominantly associated with developmental activity and to a lesser extent defines an imprinted allele with the potential to be active.

The presence of H3K4me3 and H3K27me3 together at 25% and 20% of developmentally *expressed *imprinted genes in ESCs and MEFs respectively (Figure 4) likely indicates an association of H3K27me3 with the inactive allele. H3K27me3 and H3K9me3 have been previously postulated to play a role in maintaining silencing of the inactive allele of imprinted genes without promoter DMRs in the mouse placenta [24,27]. However, we have found that H3K9me3 is not enriched at any imprinted genes without a promoter DMR in ESCs (Figure 2) or in NPCs or MEFs (*n *= 22 for both cell types; data not shown; note also that no imprinted genes with a promoter DMR are enriched for H3K9me3 in NPCs, *n *= 26). For H3K27me3, enrichment is not observed at *all *imprinted genes that do not possess a promoter DMR, but rather at only 55% of these genes in ESCs (Figure 2), confirming that this modification does not always mark the inactive allele in the absence of DNA methylation. In NPCs and MEFs, H3K27me3 enrichment is found at only 32% and 45% of genes without promoter DMRs respectively (*n *= 22 for both cell types; data not shown). Furthermore, when assessing only imprinted genes that do not have a promoter DMR and are developmentally expressed in ESCs (in order to exclude H3K27me3 enrichment involved in developmental repression), H3K27me3 is enriched only 20% of the time (*n *= 10; data not shown); H3K27me3 enrichment is found at 30% of developmentally expressed imprinted genes that do possess a promoter DMR (*n *= 10). This implies that H3K27me3 does not commonly repress the inactive allele in the absence of differential promoter methylation in mouse ESCs and that enrichment is not generally dependent on promoter DMR status. No developmentally expressed genes are enriched with H3K27me3 in NPCs; in MEFs an equal level of enrichment is observed at imprinted genes with promoter DMRs (20%, *n *= 10) and without promoter DMRs (20%, *n *= 10) of those that are developmentally expressed.

## Discussion

The comparative characterisation of histone modification profiles, DMR status and expression profiles for all confirmed imprinted genes provides a valuable resource for those interested in the role of these epigenetic marks in different classes of gene expression and repression and in the regulation of genomic imprinting in particular. Additionally, patterns of histone modification enrichment common to imprinted genes have been identified and highlight differences in the epigenetic profiles of these particular genes compared to other genes. In our analyses, we have been able to infer imprinted rather than developmental repression through the identification of modifications always associated with a germline DMR regardless of the developmental expression status of the gene. Histone modification enrichment profiles found to associate with the developmental expression state are acknowledged to be involved in the developmental regulation of imprinted genes.

### Repressive marks associated with imprinting regulation

The repressive marks H3K9me3 and H4K20me3 are unlikely to play a prominent role in developmental repression of imprinted genes. This is apparent through the finding that H3K9me3 and H4K20me3 are not enriched at developmentally repressed genes any more often than at expressed genes in mouse ESCs (Additional file 2). Instead, these two marks are exclusively enriched at imprinted genes possessing promoter DMRs, the majority of which are germline DMRs, consistent with imprinting control (Figure 2). Based on these results we conclude that H3K9me3 and H4K20me3 are associated with imprinting repression rather than developmental repression.

There are two features of H3K27me3 that we have explored: that of H3K27me3 enrichment at inactive imprinted alleles and as part of a bivalent mark at the normally active imprinted allele involved in developmental repression (the latter is discussed subsequently). The presence of H3K27me3 at up to a quarter of developmentally expressed imprinted genes in both mouse ESCs and MEFs (Figure 4) implies that this repressive histone mark may, in some cases, associate with the inactive imprinted allele, indicating involvement in imprinting control.

Some inactive imprinted alleles are associated with DNA methylation and others are not. As we have identified H3K27me3 to be involved in imprinting regulation at some genes, we investigated whether this histone mark might play a role in repressing the inactive allele in the absence of DNA methylation, as suggested by others [24]. Several previous studies have found that H3K27me3 does indeed mark the inactive imprinted allele in the absence of DNA methylation [10,12,24,32,42]. In contrast, other findings have reported H3K27me3 to be preferentially enriched on the inactive DNA methylated allele at some imprinted genes with a promoter DMR [10,12,16,24,25,35,39,41,42]. Our results show that H3K27me3 enrichment is not widely dependent on the presence or absence of differential DNA methylation at the promoter of an imprinted gene (Figure 2). In addition, H3K9me3 never marks the inactive allele of an imprinted gene in the absence of a promoter DMR in mouse ESCs, NPCs or MEFs (Figure 2 and data not shown). Therefore, as neither of these two repressive histone marks consistently correlate with the absence of DNA methylation at the inactive imprinted allele, other as yet unidentified epigenetic configurations or protein complexes may hold the inactive allele in a repressive state when no DMR is present.

### A role for H3K27me3 in developmental repression of imprinted genes

The histone mark H3K27me3 is more often found at developmentally repressed than at expressed imprinted genes in mouse ESCs (see Additional file 2) demonstrating that H3K27me3 likely plays a prominent role in the developmental repression of imprinted genes. Furthermore, in NPCs and MEFs, higher enrichment of H3K27me3 alone is observed at developmentally repressed compared to expressed imprinted genes (Figure 4). Although this mark has been previously examined at many imprinted genes, few studies have looked in tissues where the gene is developmentally repressed. Three studies provide independent support for the above finding. The first example is from a study of the *Igf2r*/*Airn *imprinted locus. In mouse fibroblasts where *Slc22a2 *and *Slc22a3 *are developmentally (and hence biallelically) repressed, widespread H3K27me3 was enriched on both parental chromosomes [20]. Yamasaki-Ishizaki *et al*. [32] assessed the relative histone modifications present on each allele at the major-type promoter of the mouse imprinted gene *Grb10*. They also established that H3K27me3 marks both chromosomes in neurons where this transcript is developmentally repressed. At the brain-type promoter of *Grb10*, Sanz *et al*. [38] found that H3K27me3 marks the 'normally active' allele in non-neuronal tissues where this alternative transcript is developmentally repressed. Our results imply that developmental repression of the normally active allele of an imprinted gene by H3K27me3 is a much more widespread phenomenon than just these examples and suggests that imprinted genes are developmentally repressed by this mark in a similar manner to non-imprinted genes.

### Bivalency at imprinted genes

The modifications H3K4me3 and H3K27me3 are commonly found together at imprinted genes in both mouse and human ESCs, as described above and illustrated in Figure 1. The results of our additional analyses in mouse suggest that these two modifications are often (although not always) found on the normally active allele when it is developmentally repressed.

Firstly, we find H3K4me3 enrichment at a high proportion of developmentally repressed imprinted genes in mouse ESCs, which most likely reflects enrichment on the 'normally active' allele (Additional file 2). This is independently validated by previous studies in both mouse and human which have shown enrichment of H3K4me3 or H3K4me2 (a mark also associated with bivalent domains [61]) on the normally active alleles of imprinted genes in cell types where the gene is developmentally, hence biallelically, repressed [7,15,31,32]. Additionally, H3K27me3 is associated with developmental repression of imprinted genes as discussed above. We also observe a reduction in the number of genes enriched with both H3K4me3 and H3K27me3 in more differentiated cell types compared to ESCs, with a corresponding increase in genes enriched with only one of these marks, suggestive of bivalency resolution (Figure 3; also see Table 1). Lastly, the presence of H3K4me3 and H3K27me3 together at repressed imprinted genes more often than at expressed genes in ESCs, and also to some degree in NPCs and MEFs, (Figure 4) implies a role for this combinatorial profile in developmental repression. Hence, in some cases, this profile regulates the developmentally controlled allele. This suggests that a number of developmentally repressed imprinted genes exhibit bivalency on the normally active allele. Direct support for this is shown by two previous allele-specific studies identifying H3K27me3 preferentially enriched on the normally active, but developmentally repressed paternal allele of the *Grb10 *brain-type promoter transcript along with H3K4me2, characteristic of a bivalent domain [38,40].

Many examples of developmental regulation through bivalency have been published at non-imprinted genes (reviewed in [62]). Studies first identifying the bivalent state were performed in ESCs and this state was initially suggested to hold genes in a repressive yet primed or poised manner in order to enable rapid activation upon receipt of developmental cues [58,61]. However, further studies have identified the acquisition of this profile at genes that become repressed and at genes in more differentiated cell types (for example, [50]). The polycomb group complex may represent a mechanism allowing rapid, flexible gene regulation during development. Many imprinted genes have dynamic expression profiles during development [63], therefore it is perhaps not surprising that they may commonly show bivalency.

### Tri-mark epigenetic signature at imprinting control regions

Our analyses show that all ICRs display enrichment of H3K9me3 and H4K20me3 in combination with H3K4me3, constituting an epigenetic signature. Previous reports have identified enrichment of H3K27me3 on the methylated chromosome of several ICRs [6,24,25,35,41], however, we have not found enrichment of this mark at all known ICRs. This implies that H3K27me3 is not a consistent epigenetic feature of these primary imprinting control elements, unlike the three aforementioned modifications. Hybrid ESCs (generated from a mouse cross between different genetic backgrounds) were employed by Mikkelsen *et al*. [50] to identify differences in histone modification enrichment between the two parental chromosomes. H3K4me3 was found enriched to a greater degree on the unmethylated than the methylated chromosome at a number of ICRs, however there were not enough informative reads for H3K9me3 and H4K20me3 was not tested. These repressive marks are nonetheless most likely preferentially enriched on the methylated chromosome of ICRs and several previous studies support this [6,20,28,35,36,38]. Pericentric heterochromatin is marked by DNA methylation, H3K9me3 and H4K20me3 (reviewed in [64]), which is identical to the epigenetic profile identified on the methylated chromosome of both promoter and intergenic ICRs. This suggests that epigenetic repression at these primary control elements is more similar to heterochromatin repression than to developmental gene silencing. Regulatory mechanisms are not identical, however, as it has previously been reported that deposition of H3K9me3 is controlled at some ICRs by G9a and SETDB1 rather than the methyltransferase SUV39H which is employed at heterochromatin [20,27,36].

## Conclusions

**Figure 5:**
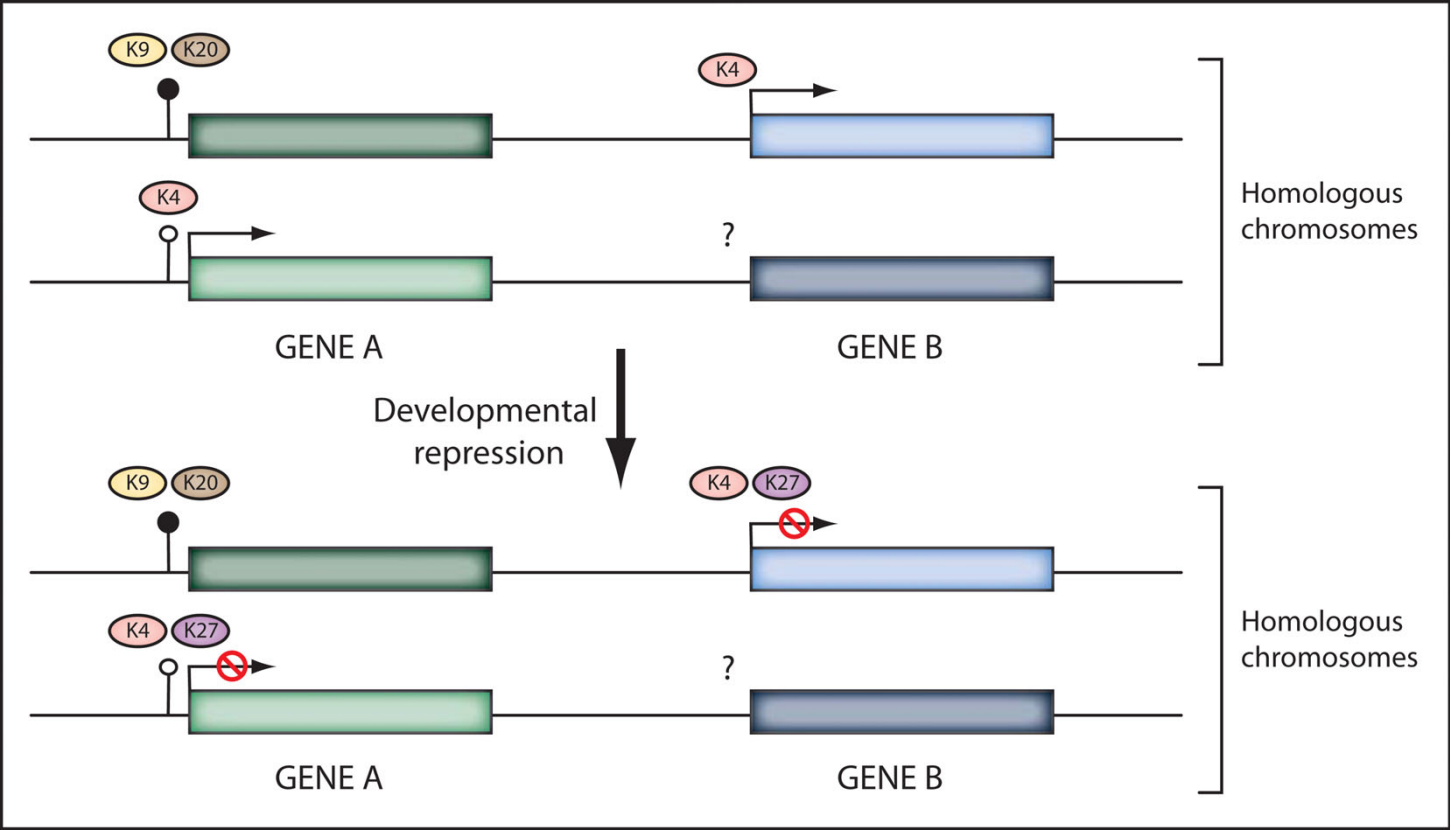
**Working model of histone modification profiles at imprinted genes undergoing developmental repression**. In mouse embryonic stem cells, the presence of a germline differentially methylated region reflecting an imprinting control region at the promoter of gene A confers a distinct histone modification profile to that of gene B, which has no differential DNA methylation; gene A is marked by H3K4me3 on the active allele and by H3K9me3 plus H4K20me3 on the inactive allele. After developmental repression, both genes acquire H3K27me3 on the previously active allele. Transcriptional activators and repressors are key players in this process and may be a cause or consequence of the histone modification states shown. All histone modifications illustrated represent the trimethylated state.

The analyses performed here provide insight into the association of histone modifications with different forms of genomic regulation. In all the cell types assessed, H3K27me3 is frequently found associated with developmental repression of imprinted genes, whereas H3K9me3 and H4K20me3 correlate with repression of the inactive allele of imprinted genes possessing promoter DMRs, rather than with spatiotemporally controlled developmental repression in ESCs. H3K4me3 is found associated with developmental regulation. We further propose that bivalent domains act to developmentally repress imprinted genes, as they do for other genes in the genome. In addition, we have identified an H3K4me3, H3K9me3 and H4K20me3 tri-mark signature at all ICRs without exception in mouse ESCs. Figure 5 illustrates a working model of the epigenetic marks associated with imprinted and developmental gene regulation.

It is demonstrated here that high-throughput studies can be mined and analysed for smaller functional categories to generate novel insights into different forms of epigenetic regulation. Through genome-wide analysis, we have shown that alleles inactivated by genomic imprinting can be distinguished epigenetically from developmentally repressed alleles. These findings are consistent with published allele-specific data on individual imprinted loci (see Additional file 3) which therefore provides independent validation of our results. Our findings suggest that the germline mark crucial for the establishment of genomic imprinting is specifically acted upon, at least in ESCs, by different repressive epigenetic mechanisms than those regulating more canonical developmental repression.

## Methods

All mouse and human imprinted genes confirmed at the time of analysis [52-54] were assessed for their respective histone modification enrichment profiles through computational analysis of publicly available data. Official Mouse Genome Informatics (MGI) symbols, RefSeq accession numbers and alias gene names were utilised to pull data specific to imprinted genes from source files containing whole-genome data from six high-throughput studies [49-51,55-57] using the grep command line utility. This data was then analysed for individual and combinatorial histone modification enrichment profiles. Incorporation of expression status and promoter DMR status was subsequently undertaken.

Source data files contained genomic regions classified by the respective authors as enriched with a particular histone modification or not. Enrichment at TSSs was determined by intersecting genomic coordinates using the program Galaxy [65] when not provided.

A number of imprinted genes were absent in data files from all six studies; this is most likely due to the unconfirmed status of many imprinted genes in current reference gene databases. The imprinted non-coding RNAs *Airn*, *Kcnq1ot1 *and *Nespas *were not included in source data files from any study, therefore the histone modification profiles were manually added using original enrichment data from Mikkelsen *et al*. [50]. Genomic coordinates of enrichment for H3K4me3, H3K27me3, H3K9me3 and H4K20me3 were assessed for overlap with the TSS coordinates of these genes.

Distinct experimental methods were performed by different laboratories to assess developmental gene expression status genome-wide. Expression status is based on GIS-PET evidence for Zhao *et al*. [51], while for Guenther *et al*. [49] expression is characterised in Additional file 1: Chromatin and expression states when data pooled from microarray experiments and massively parallel signature sequencing (MPSS) experiments were consistent. For microarrays performed by Mikkelsen *et al*. [50], we define a gene as repressed if the microarray signal intensity value is below 25.

All six high-throughput studies were mined to characterise histone modification enrichment and expression status at all confirmed mouse and human imprinted genes as described above and depicted in Additional file 1: Chromatin and expression states. The data of Mikkelsen *et al*. [50] alone was subsequently analysed to maintain consistency between species, cell types and experimental techniques. These authors used two methods for defining a genomic region as enriched with a particular histone modification. Enriched regions for H3K4me3, H3K27me3 and H3K9me3 were defined using a Window Interval (WI) method for all cell types while for H4K20me3, a Hidden Markov Model (HMM) methodology was employed. As analysis using the HMM was also undertaken by the authors for H3K4me3, H3K27me3 and H3K9me3 in ESCs only, we characterised the number of imprinted genes enriched with these histone modifications using both methods to assess their comparability. 1.9%, 1.9% and 5.6% of imprinted genes differed in classification of enrichment for H3K4me3, H3K27me3 and H3K9me3 respectively between the two methods. We therefore assessed these three marks using the WI method to allow direct comparison between cell types, whereas H4K20me3 enrichment was assessed using the HMM method which may result in a slight underrepresentation in comparison.

### Statistical analyses

We have undertaken chi-square tests using Yates' correction to statistically evaluate single histone modification profiles under different conditions. Chi-square contingency tables were used when comparing multiple histone modification profiles under different conditions.

## Abbreviations

DMR: differentially methylated region; ESC: embryonic stem cell; HMM: Hidden Markov Model; ICR: imprinting control region; MEF: mouse embryonic fibroblast; MGI: Mouse Genome Informatics; MPSS: massively parallel signature sequencing; NPC: neural progenitor cell; REH: human pro-B cells; TSS: transcription start site; WI: Window Interval.

## Competing interests

The authors declare that they have no competing interests.

## Authors' contributions

AFS and KM designed the study, KM conducted the analysis and generated the data. KM and AFS interpreted the data and wrote the paper.

## Supplementary Material

Additional file 1 **Imprinted gene characterisation**. Imprinted genes: this worksheet lists the imprinted genes mined for histone modification and expression status from source data of high-throughput papers; Promoter differentially methylated region (DMR) status: this worksheet details the promoter DMR status of imprinted genes used for analysis of histone modification profiles at genes with and without promoter DMRs, including references; Chromatin and expression states: this worksheet provides data extracted from six high-throughput studies for all available imprinted genes. Notably, not all imprinted genes were present in the source data files, most likely due to the high proportion of imprinted genes currently holding a predicted status in gene reference databases. If a gene was not present in one source data file the output is given as 'Gene not present'. Genes imprinted only in mice, or where imprinting status is not confirmed in humans, or where no orthologous human gene exists (*), are excluded from further human analyses. The same applies for human-specific imprinted genes (^†^), which are excluded from all mouse analyses. Isoform-dependent imprinted genes (^‡^) are not included in any further analyses. hESC, human embryonic stem cells; mESC, mouse embryonic stem cells; REH, human pro-B cells; mNPC, mouse neural progenitor cells; MEF, mouse embryonic fibroblasts; TSS, transcription start site; HCP, high CpG promoter; ICP, intermediate CpG promoter; LCP, low CpG promoter; MPSS, massively parallel signature sequencing. Click here for file

Additional file 2 **Histone modification enrichment at developmentally expressed and repressed imprinted genes in embryonic stem cells (ESCs)**. Enrichment of H3K4me3, H3K27me3, H3K9me3 and H4K20me3 at transcription start sites of developmentally expressed and repressed imprinted genes were assessed in mouse ESCs using high-throughput enrichment and expression source data from Mikkelsen *et al*. [50]. The presence of one particular modification at an imprinted gene does not preclude the presence of another. Developmentally expressed and developmentally repressed imprinted genes do not have significantly different epigenetic profiles for these four marks (chi-square contingency test, *P *= 0.525). Click here for file

Additional file 3 **Allele-specific histone modification enrichment at imprinted genes**. In order to compare the results of our genome-wide analyses with allele-specific data, we have characterised previously published histone modification enrichment profiles involved in imprinting and developmental repression at imprinted genes. This data supports our findings and provides independent validation of our results. Click here for file
