# Supplementary material for: Distinguishing epigenetic marks of developmental and imprinting regulation
Source: Epigenetics Chromatin. 2010 Jan 15;3:2. doi: 10.1186/1756-8935-3-2 (PMC2841594; doi:10.1186/1756-8935-3-2)
Supplement: Additional file 3 — Allele-specific histone modification enrichment at imprinted genes. In order to compare the results of our genome-wide analyses with allele-specific data, we have characterised previously published histone modification enrichment profiles involved in imprinting and developmental repression at imprinted genes. This data supports our findings and provides independent validation of our results. [file 1756-8935-3-2-S3.PDF]

	Histone differential enrichment profile	Gene(s)	References
Imprinting repression	H3K27me3 at inactive allele	*Ascl2*, *Cd81*, *Cdkn1c*, *Grb10 major-type*, *H19*, *Igf2*, *Kcnq1*, *Kcnq1ot1*, *Osbpl5*, *Peg10*, *Phlda2*, *Snrpn*, *Tssc4*	[1-11]
	H3K9me3 at inactive allele	*Air*, *Ascl2*, *Cd81*, *Cdkn1c*, *Gnas Exon1A*, *Gnasxl*, *Grb10 brain-type*, *H19*, *Igf2r*, *Kcnq1ot1*, *Nesp*, *Nespas*, *Peg10*, *Snrpn*, *Zrsr1*	[6, 9-20]
	H4K20me3 at inactive allele	*Air*, *Grb10 brain-type*, *Kcnq1ot1*, *Peg10*, *Snrpn*	[6, 11, 15, 17-20]
Developmental repression	H3K27me3 without H3K4me3 at developmentally repressed allele	*Grb10 major-type*, *Slc22a2*, *Slc22a3*	[10, 15]
	H3K4me2/3 at developmentally repressed allele	*Air*, *Grb10 brain-type*, *NDN*, *Snrpn*, *Zrsr1*	[10, 14, 21, 22]
	H3K4me2 and H3K27me3 at developmentally repressed allele	*Grb10 brain-type*	[19, 20]

1. Lewis A, Mitsuya K, Umlauf D, Smith P, Dean W, Walter J, Higgins M, Feil R, Reik W: **Imprinting on distal chromosome 7 in the placenta involves repressive histone methylation independent of DNA methylation.** *Nat Genet* 2004, **36** (12): 1291-1295.
2. Lewis A, Green K, Dawson C, Redrup L, Huynh KD, Lee JT, Hemberger M, Reik W: **Epigenetic dynamics of the Kcnq1 imprinted domain in the early embryo.** *Development* 2006, **133** (21): 4203-4210.
3. Han L, Lee DH, Szabo PE: **CTCF is the master organizer of domain-wide allele-specific chromatin at the H19/Igf2 imprinted region.** *Mol Cell Biol* 2008, **28** (3): 1124-1135.
4. Umlauf D, Goto Y, Cao R, Cerqueira F, Wagschal A, Zhang Y, Feil R: **Imprinting along the Kcnq1 domain on mouse chromosome 7 involves repressive histone methylation and recruitment of Polycomb group complexes.** *Nat Genet* 2004, **36** (12): 1296-1300.
5. Monk D, Arnaud P, Apostolidou S, Hills FA, Kelsey G, Stanier P, Feil R, Moore GE: **Limited evolutionary conservation of imprinting in the human placenta.** *Proc Natl Acad Sci U S A* 2006, **103** (17): 6623-6628.
6. Monk D, Wagschal A, Arnaud P, Muller PS, Parker-Katiraee L, Bourc'his D, Scherer SW, Feil R, Stanier P, Moore GE: **Comparative analysis of human chromosome 7q21 and mouse proximal chromosome 6 reveals a placental-specific imprinted gene, TFPI2/Tfpi2, which requires EHMT2 and EED for allelic-silencing.** *Genome Res* 2008, **18** (8): 1270-1281.
7. Terranova R, Yokobayashi S, Stadler MB, Otte AP, van Lohuizen M, Orkin SH, Peters AH: **Polycomb group proteins Ezh2 and Rnf2 direct genomic contraction and imprinted repression in early mouse embryos.** *Dev Cell* 2008, **15** (5): 668-679.
8. Kim JM, Ogura A: **Changes in allele-specific association of histone modifications at the imprinting control regions during mouse preimplantation development.** *Genesis* 2009, **47** (9): 611-616.
9. Verona RI, Thorvaldsen JL, Reese KJ, Bartolomei MS: **The transcriptional status but not the imprinting control region determines allele-specific histone modifications at the imprinted H19 locus.** *Mol Cell Biol* 2008, **28** (1): 71-82.

10. Yamasaki-Ishizaki Y, Kayashima T, Mapendano CK, Soejima H, Ohta T, Masuzaki H, Kinoshita A, Urano T, Yoshiura K, Matsumoto N, Ishimaru T, Mukai T, Niikawa N, Kishino T: **Role of DNA methylation and histone H3 lysine 27 methylation in tissue-specific imprinting of mouse Grb10.** *Mol Cell Biol* 2007, **27** (2): 732-742.
11. Delaval K, Govin J, Cerqueira F, Rousseaux S, Khochbin S, Feil R: **Differential histone modifications mark mouse imprinting control regions during spermatogenesis.** *Embo J* 2007, **26** (3): 720-729.
12. Vu TH, Li T, Hoffman AR: **Promoter-restricted histone code, not the differentially methylated DNA regions or antisense transcripts, marks the imprinting status of IGF2R in human and mouse.** *Hum Mol Genet* 2004, **13** (19): 2233-2245.
13. Li T, Vu TH, Ulaner GA, Yang Y, Hu JF, Hoffman AR: **Activating and silencing histone modifications form independent allelic switch regions in the imprinted Gnas gene.** *Hum Mol Genet* 2004, **13** (7): 741-750.
14. Fournier C, Goto Y, Ballestar E, Delaval K, Hever AM, Esteller M, Feil R: **Allele-specific histone lysine methylation marks regulatory regions at imprinted mouse genes.** *Embo J* 2002, **21** (23): 6560-6570.
15. Regha K, Sloane MA, Huang R, Pauler FM, Warczok KE, Melikant B, Radolf M, Martens JH, Schotta G, Jenuwein T, Barlow DP: **Active and repressive chromatin are interspersed without spreading in an imprinted gene cluster in the mammalian genome.** *Mol Cell* 2007, **27** (3): 353-366.
16. Wagschal A, Sutherland HG, Woodfine K, Henckel A, Chebli K, Schulz R, Oakey RJ, Bickmore WA, Feil R: **G9a histone methyltransferase contributes to imprinting in the mouse placenta.** *Mol Cell Biol* 2008, **28** (3): 1104-1113.
17. Wu MY, Tsai TF, Beaudet AL: **Deficiency of Rbbp1/Arid4a and Rbbp1l1/Arid4b alters epigenetic modifications and suppresses an imprinting defect in the PWS/AS domain.** *Genes Dev* 2006, **20** (20): 2859-2870.
18. Pannetier M, Julien E, Schotta G, Tardat M, Sardet C, Jenuwein T, Feil R: **PR-SET7 and SUV4-20H regulate H4 lysine-20 methylation at imprinting control regions in the mouse.** *EMBO Rep* 2008, **9** (10): 998-1005.
19. Sanz LA, Chamberlain S, Sabourin JC, Henckel A, Magnuson T, Hugnot JP, Feil R, Arnaud P: **A mono-allelic bivalent chromatin domain controls tissue-specific imprinting at Grb10.** *EMBO J* 2008, **27** (19): 2523-2532.
20. Monk D, Arnaud P, Frost J, Hills FA, Stanier P, Feil R, Moore GE: **Reciprocal imprinting of human GRB10 in placental trophoblast and brain: evolutionary conservation of reversed allelic expression.** *Hum. Mol. Genet.* 2009, **18** (16): 3066-3074.
21. Yamasaki Y, Kayashima T, Soejima H, Kinoshita A, Yoshiura K, Matsumoto N, Ohta T, Urano T, Masuzaki H, Ishimaru T, Mukai T, Niikawa N, Kishino T: **Neuron-specific relaxation of Igf2r imprinting is associated with neuron-specific histone modifications and lack of its antisense transcript Air.** *Hum Mol Genet* 2005, **14** (17): 2511-2520.
22. Lau JC, Hanel ML, Wevrick R: **Tissue-specific and imprinted epigenetic modifications of the human NDN gene.** *Nucleic Acids Res* 2004, **32** (11): 3376-3382.
